# Significance of likes: Analysing passive interactions on Facebook during campaigning

**DOI:** 10.1371/journal.pone.0179435

**Published:** 2017-06-16

**Authors:** Mohammad Adib Khairuddin, Asha Rao

**Affiliations:** 1 Mathematical and Geospatial Sciences, School of Science, RMIT University, Melbourne, VIC, Australia; 2 Jabatan Sains Komputer, FSTP, Universiti Pertahanan Nasional Malaysia, Kuala Lumpur, Malaysia; Nanyang Technological University, SINGAPORE

## Abstract

With more and more political candidates using social media for campaigning, researchers are looking at measuring the effectiveness of this medium. Most research, however, concentrates on the bare count of likes (or twitter mentions) in an attempt to correlate social media presence and winning. In this paper, we propose a novel method, Interaction Strength Plot (IntS) to measure the passive interactions between a candidate’s posts on Facebook and the users (liking the posts). Using this method on original Malaysian General Election (MGE13) and Australian Federal Elections (AFE13) Facebook Pages (FP) campaign data, we label an FP as performing well if both the posting frequency and the likes gathered are above average. Our method shows that over 60% of the MGE13 candidates and 85% of the AFE13 candidates studied in this paper had under-performing FP. Some of these FP owners would have been identified as popular based on bare count. Thus our performance chart is a vital step forward in measuring the effectiveness of online campaigning.

## Introduction

Many researchers have used the number of likes and twitter mentions as a means of determining the popularity of candidates in an election campaign. This paper will show that this sort of bare count is insufficient and the result of the subsequent analysis could be misleading. We propose a novel method for charting the interactions between a candidate’s Facebook Page (FP), and the users who like the page. Instead of just looking at the popularity of the FP, we view the interactions as a measure of performance. Plotting the interactions between the posts and the users presents an overview of the online campaigning performance of the candidates, in particular of the campaign activities posted on their FP. As our analysis will show, a well-performing FP should be defined as one that consistently demonstrates increasing and positive interactions by regularly posting at least the average number of posts for the day, with these posts gathering at least the average number of likes for the day. We believe that this ranking would be a much better indicator of the performance of an FP in campaigning than just the bare count of likes gathered.

Until now, the majority of the quantitative analyses on the use of social media in campaigning has concentrated on Twitter data and in particular, the bare counts of tweets. Among others, Tumasjan et al. [1] conclude that the tweets’ count could have been used to forecast success in the 2009 German national election. On the other hand Borondo et al. [2] use the slopes of the time series of the accumulated Twitter mentions to measure the support for each candidate in the 2011 Spanish Presidential elections. However, going one step further, they analyse the retweet and mention networks using complex network analysis to conclude that there was an absence of debate between the different political parties. While applying similar measurements, Caldarelli et al. [3] noted that even though tweets can be an effective indicator for election outcomes, the accuracy of the prediction depends on the volume of the tweets. Other researchers such as Aragón et al. [4] conclude that during the 2011 Spanish national elections, the volume of tweets show evidence of a strong correlation between the activity in Twitter and the offline campaign events.

In this paper, we look at campaigning on Facebook, since, unlike Twitter, Facebook is known to be a more diffuse social network [5] as well as a popular mass media tool [6]. FP, as one of the tools offered by Facebook, may only be created and managed by official representatives [7], and it allow organisations, businesses, celebrities and brands to communicate broadly with people who like the page. In general, campaigning on FP starts out with the owner of the FP (in this case the candidate) posting a campaign message on the FP. This message can either be an event, news, photo or video. Users who have liked the FP (or page) will be automatically notified of the posts, and they can respond to the post either by liking or commenting on the post. Interaction occurs whenever the user responds to the post posted by the candidate, and is a reactive communication, defined by Rafaeli and Sudweeks [8] to be a process whereby one side responds to the other side. We define the act of a user liking a post as a passive interaction.

The simplest method of measuring FP interactions is by counting the number of likes acquired by a candidate’s posts as has been done by many authors. These studies conclude that the number of likes acquired by a candidate during the campaign period can be used to illustrate the extent of support gained by the candidate. For example, Giglietto [5] uses the number of likes received by a candidate’s Facebook (FB) as an indicator of popularity and utilises it to prove a correlation with the outcome of the 2011 Italian municipal elections. Giglietto [5] states that it is pretty likely that the most popular candidate on FP won or came second in the election. Similarly, Barclay et al. [6] use the number of likes recorded on candidates’ FP to show a strong correlation between the number of likes and the popular vote share during the 2014 Indian Lok Sabha election. Barclay et al. [6] observe that the party that secured the most votes during the election was also the party that had the highest increment of likes (during the study period) as well as having the highest average of daily likes recorded on its FP. Both studies clearly treat the number of likes recorded on a candidate’s Facebook as an important factor in measuring the popularity of a candidate, and, in a way, assessing and predicting the candidate’s performance in the election.

We will show in this paper that this current method of solely using the number of likes recorded on FB or FP, to gauge the performance of a candidate during the campaign is not entirely accurate and does not present the whole picture. Our data shows a huge variation in the likes gained by posts, with some posts gaining vastly more likes than other posts. Thus we could say that the gaining of likes depends very much on the posts. This however, as our data shows, does not mean that a candidate needs to post a lot to gain a high number of likes. Measurements used in past studies ignore this occurrence.

The *Interaction Strength Plot* or IntS proposed in this paper aims to determine the dynamic performance of a candidate’s FP during an election campaign period, by presenting a graph that records the strength of the passive interactions between a candidate’s posts and the users’ likes across the entire duration of the campaign. IntS incorporates the variability of a candidate’s posting, as well as the probability of the posts gaining the appropriate number of likes. The methodology used in IntS is inspired by ‘Variable Life-Adjusted Display’ or VLAD [9], a control chart used mainly in the health industry, as a tool to monitor the performance of treatments and outcomes. By adapting this methodology we present a way of monitoring the performance of a candidate’s FP account.

The next section gives a brief overview of the original empirical data that we captured during the last (2013) Malaysian General Election (MGE13). This section also reviews the usage of a control chart to measure performance, together with a description of VLAD. Here we describe in detail the workings of the proposed performance measure, IntS, while the next section highlights the findings from the passive IntS (P_IntS) analysis for MGE13 data. We also compare the MGE13 P_IntS with similar data that we captured from the Australian Federal Elections held in the same year. Finally, we end the paper with the conclusion and potential future work.

## Materials and methods

### Ethics statement

For the capturing exercise, we used NodeXL [10] version 1.9.3. This version release is important as it allowed us to grab the posts and the relationships or links information based on the likes. Even though the collection period can be set on NodeXL, the captured data does not have the information on the date and time the posts are posted. In order to retrieve this information, we had to use other tools namely Microsoft Power Query for Excel [11] version 2.20 together with Facebook Graph API [12] to retrieve the date and time of posting, as well as to verify the outputs from NodeXL. All the captured data are publicly available as users with privacy restrictions are not included. We abide by the terms, conditions and privacy policies of the websites (Facebook). In addition, RMIT College Human Ethics Advisory Network (CHEAN) has exempted the research from ethics review as the captured data (FP posts and likes) used in this research are public and not from personal accounts or Facebook Timeline.

### Data

**Fig 1:**
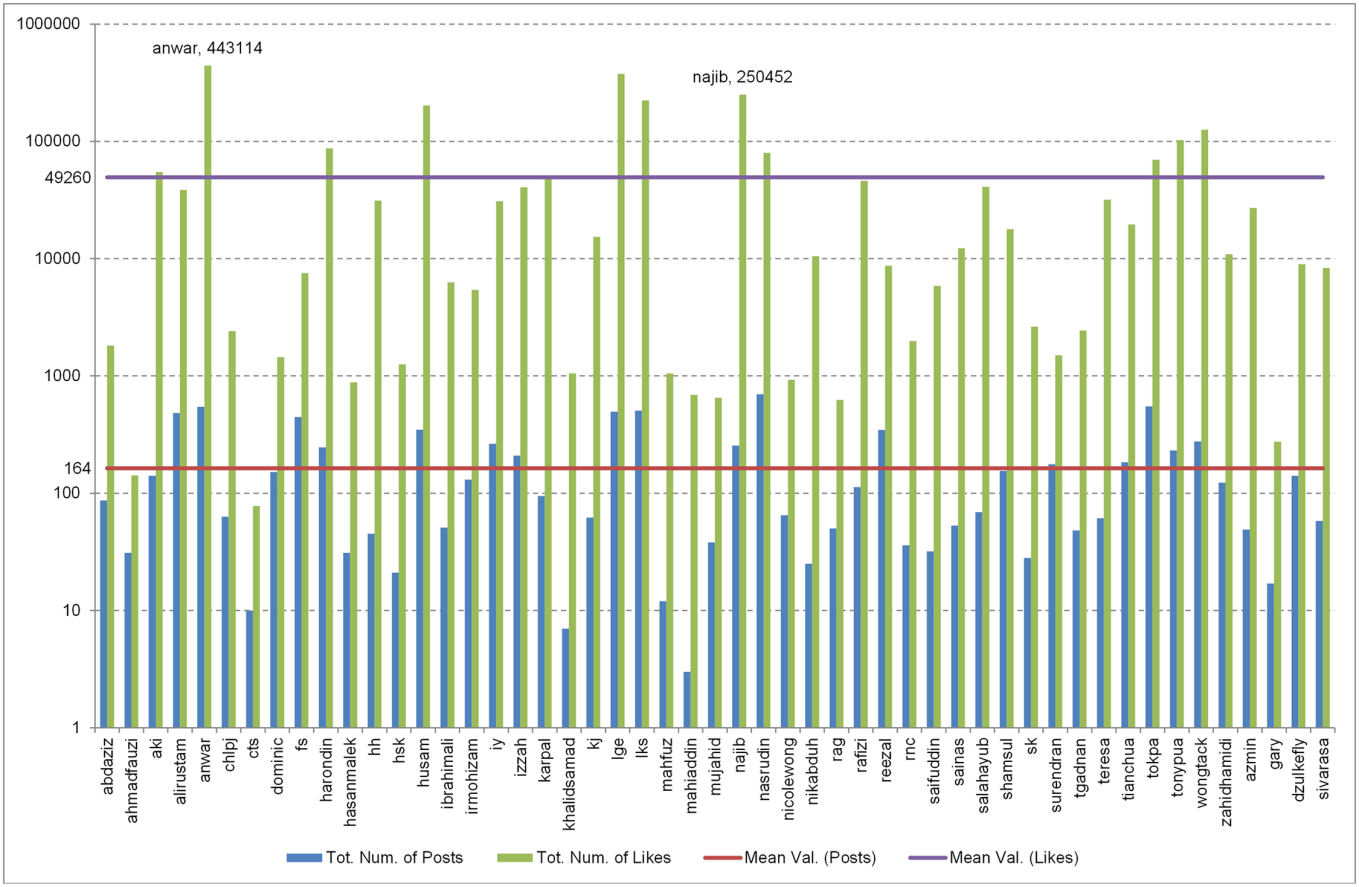
Total *Posts* and *Likes* of the MGE13 FP data. Number of posts (blue bar) and the acquired likes (green bar) recorded on the FP of 51 candidates during the MGE13 campaign. The y-axis shows the log of posts and likes of each candidate as indicated on the x-axis. The purple line indicates the average number of likes received, while the red line indicates the average number of posts published. The graph also shows the total number of likes gained by Najib Razak (*najib*) and Anwar Ibrahim (*anwar*) over the 33 days of campaigning.

The 2013 Malaysian General Elections (MGE13) data is a collection of posts and likes captured directly from 51 FP owned by 51 candidates contesting in the MGE13 election for seats in the Malaysian Parliament. Our assumption is that these FP are authorised and managed by either the candidates themselves or their campaign team, as per the statement given by [7]. The collection period covered 33 days of campaigning, beginning with the dissolution of parliament, 3rd April 2013 and ending with the voting day, 5th May 2013. In general, over the campaigning period, each candidate posted on average 164 posts, and managed to acquire on average 49,260 likes. In total, the MGE13 data contains 8,348 posts with the total number of likes acquired being 2,512,248. Fig 1 illustrates these facts.

Previous research by, for example, [5] and [6] only considers the bare counts of the likes as a measurement of popularity. Thus popular candidates are those who managed to grab the most (more than average) number of likes on their FP, and as Fig 1 reveals, 12 candidates achieved this, with Anwar Ibrahim (*anwar*) leading the group. However, 3 out of this group, Husam Musa (*husam*), Wong Tack (*wongtack*) and Haron Din (*harondin*), failed to win the election, even though their acquired likes were higher when compared to their opponents. Tengku Adnan (*tgadnan*) and Shahidan Kassim (*sk*), the opponents of Husam Musa and Haron Din respectively won fewer likes but more votes. No FP account was detected for Wong Tack’s opponent. Clearly, likes do not necessarily equate to votes.

Our belief is that even though the count of likes can be used to measure responses and in this case interactions, the importance of posts should not be ignored as is being done in existing research. Our data shows that being an active candidate, that is, by posting more posts on FP, the chances of the candidate winning the election increases. However being active is also not an assurance of winning. There are 17 FP that posted above average (≥164 posts), but out of these 17 active candidates, four, namely Ali Rustam, Husam Musa, Ibrahim Yaacob and Haron Din (FP ID: *alirustam, husam, iy* and *harondin*, respectively) lost the election to opponents who were less active, namely Shamsul Iskandar, Tengku Adnan, Ahmad Fauzi and Shahidan Kassim (FP ID: *shamsul, tgadnan, ahmadfauzi* and *sk*) respectively.

### Quantifying FP passive interactions

We give some basic statistical results on the data first. We start with the mathematical formulation of the variables.

Let *n* be the number of FP analyzed, which is also the number of candidates with an FP as an official FP used in an election campaign can only be maintained by candidates. For MGE13, *n* = 51.

Let F be the set of candidates that used FP in the election campaign,

$$\begin{array}{c} F=\{F^{i}\in F,1\leq i\leq n\}. \end{array}$$ (1)

Unlike previous research, we are interested in the posting variability of the candidates. Hence assume that during the campaign period candidate *F*^*i*^ posts some content on his FP. Thus with each *F*^*i*^, there is a set of posts. Let *t*^*i*^ = total number of posts posted by candidate *F*^*i*^.

For each *F*^*i*^, if there exist posts $P_{j}^{i}$, 1 ≤ *j* ≤ *t*^*i*^, then let

$$\begin{array}{c} P^{i}=\left\{\text{All}\;\text{posts}\;\text{posted}\;\text{by}\;\text{candidate}\;F^{i}\right\}=\left\{P_{j}^{i}:1\leq j\leq t^{i}\right\}. \end{array}$$ (2)

Next, for each FP, there exists a set of users who like a post published on the FP. Since a user can only like a post once, the set of users and the set of likes of a particular post are interchangeable, however a user could like more than one post. Let $L^{i}=\text{number}\;\text{of}\;\text{likes}\;\text{gained}\;\text{by}\;\text{all}\;\text{posts}\;\text{by}\;\text{candidate}\;F^{i}$. If $\ell_{j}^{i}=$ number of likes gained by post $P_{j}^{i}$ of candidate *F*^*i*^, then $L^{i}=\sum_{j=1}^{t^{i}}\ell_{j}^{i}$.

In addition let,

$$\begin{array}{c} P=\{P^{i}:1\leq i\leq n\}=\;\text{set}\;\text{of}\;\text{all}\;\text{posts}\;\text{by}\;\text{all}\;\text{candidates,}\;\text{and} \end{array}$$ (3)

$$\begin{array}{c} L=\sum_{i=1}^{n}L^{i}=\;\text{number}\;\text{of}\;\text{likes}\;\text{gained}\;\text{by}\;\text{all}\;\text{the}\;\text{candidates.} \end{array}$$ (4)

### MGE13 regression analysis

**Fig 2:**
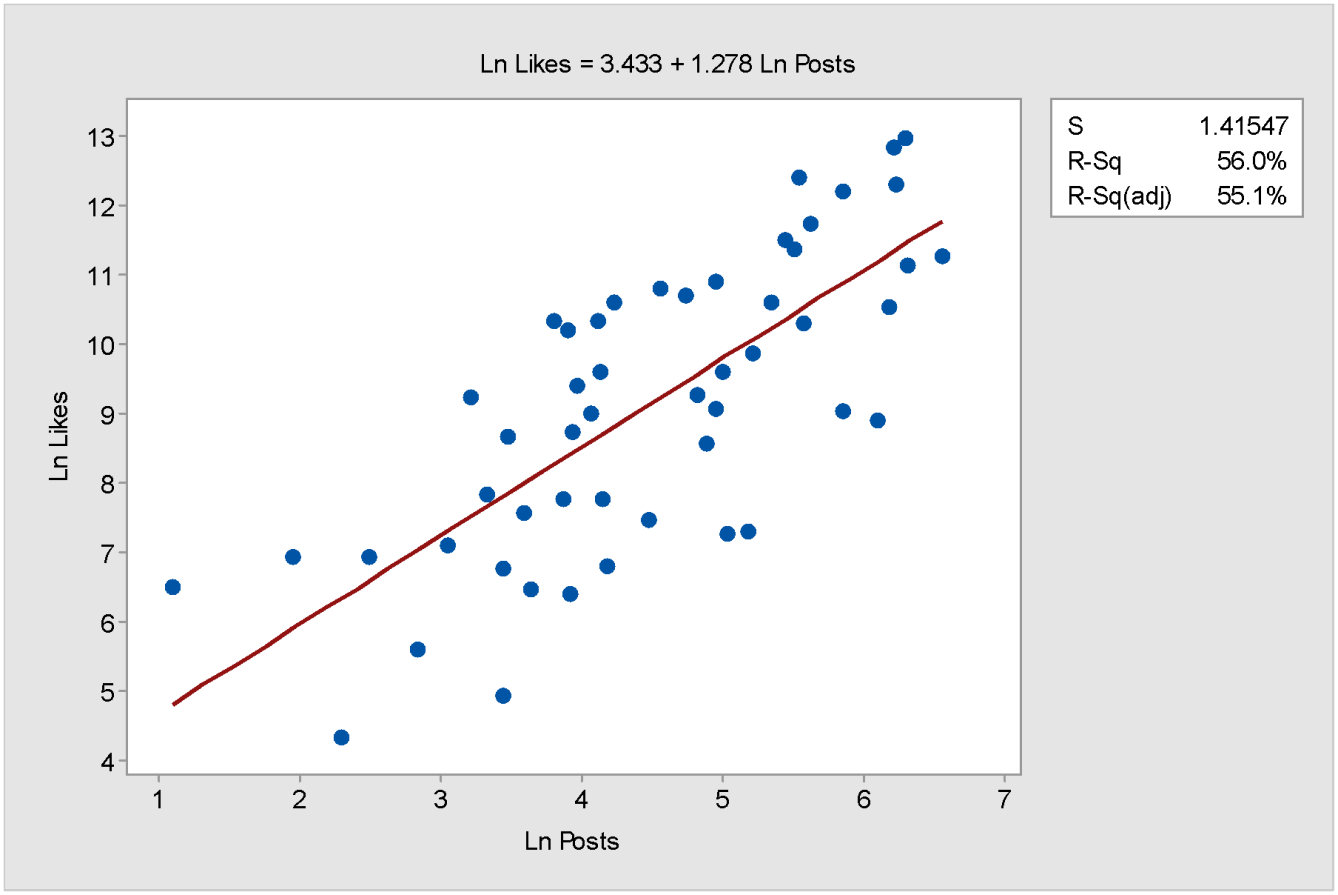
MGE13 Linear Regression (Ln). Scatterplot with Linear Regression Line of Ln Likes ($L^{i}$) vs Ln Posts ($P^{i}$) for MGE13 data.

We start our analysis by looking at the regression model, often used to examine and understand the correlation between the variables, in this case posts and acquired likes, in more detail. A good indicator of the relationship between $P^{i}$ the independent variable and $L^{i}$ the dependent variable is obtained by plotting the natural log of both variables, presented in Fig 2. The points on the graph are dispersed, balanced and near to the regression line. Pearson correlation suggests that the correlation between ln likes and ln posts is strong, at 0.749. The regression model of ln $L^{i}$ versus ln $P^{i}$ indicates that the relationship is statistically significant, *p* < 0.0005, and 56% of the variation in ln $L^{i}$ can be explained.

The regression equation for the model presented in Fig 2 is

$$\begin{array}{c} \text{Ln}\;L^{i}=3.433+1.278\;\text{Ln}\;P^{i}, \end{array}$$ (5)

with the value of $L^{i}$ given by, $L^{i}=e^{3.433}*{P^{i}}^{1.278}$.

The model shows that the value of $L^{i}$ will increase whenever the value of $P^{i}$ increases. In other words, if the candidate posted just one post, *P*^*i*^ = 1, the predicted $L^{i}$ acquired would be,

$$\begin{array}{c} L^{i}=e^{3.433}*{\left(1\right)}^{1.278}=30.97\approx 31\;\text{likes.}\; \end{array}$$ (6)

**Fig 3:**
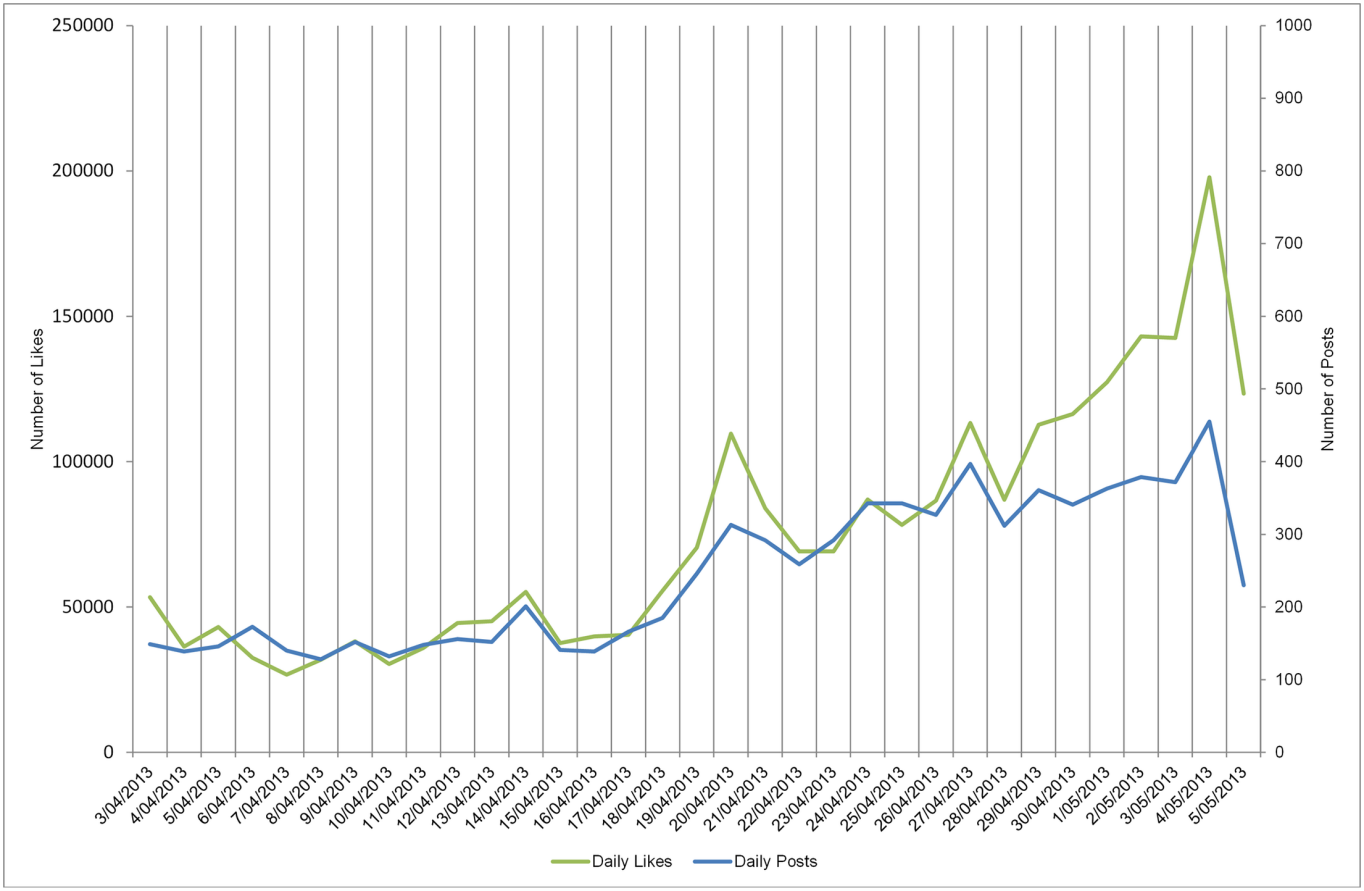
MGE13 daily *Posts* and *Likes*. Line graph of the number of posts (blue line) with the acquired number of likes (green line) for every day over the period of the MGE13 campaign.

The value of 31 likes for a post is generally an ideal case and, as can be seen from the scatterplot (Fig 2), many posts gained many more likes, while other posts gained far fewer. Fig 3 shows that over the period of campaigning there was much variation in the ability of the posts to attract likes, with posts on certain days able to attract many more likes than on other days.

Keeping these factors in mind, in this paper we explore a different way of measuring and illustrating the performance of an FP with regards to passive interactions (likes).

### Measuring performance using control chart

The use of a control chart to detect small persistent process changes has been widely practiced in many industrial production processes [13]. Coory et al. [14] state that a control chart provides an understandable and up-to-date overview of runs of good or bad outcomes, and the ability to detect problems early. One of the common control charts used to illustrate changes in performance is the cumulative sum plots or CUSUM chart. Woodall and Montgomery [15] reviewing this as one of the applications of statistical process monitoring conclude that CUSUM is suitable for a collection of data over time as it provides a quick detection of specified process changes and includes an in-control performance metric such as false-alarm rate. According to Steiner et al. [16], CUSUM is a well-established sequential monitoring scheme designed to detect changes in a process. By including estimated risk specific to each individual case into the adjustment of the CUSUM plot, Steiner et al. [16] show that the use of risk-adjusted CUSUM (RA CUSUM), has given the health industries a logical and quantified way of detecting and monitoring performance, especially risk-adjusted binary events. However, setting appropriate thresholds and boundaries to be used in RA CUSUM is quite difficult and not at all a straightforward process [17].

As an alternative to the complexity of RA CUSUM, ‘Variable Life-Adjusted Display’ or VLAD is a simpler method used by the health industry to monitor performance over a range of treatments and outcomes [9]. It is a graphical technique that incorporates information on estimated risk for each individual case [18] and can be used for any binary short-term outcome [19]. In relation to cardiac surgery, Lovegrove et al. [9] describe VLAD as a plot that shows the difference between the cumulative expected mortality and the deaths that actually occurred, taking into account the expected risk (baseline probability) associated with the particular caseload. Every case in the series is plotted from left to right on a horizontal axis, and moving across, the line moves up for survivors and down for deaths. Sherlaw [20] formulates that if *X*_*n*_ denotes the outcome for the *n*^th^ patient, and *y*_*n*_ the corresponding risk, VLAD can be calculated as,

$$\begin{array}{c} V_{n}=\sum_{i=1}^{n}y_{n}-\sum_{i=1}^{n}X_{n}. \end{array}$$ (7)

Overall, VLAD is designed to provide qualitative information [20] and can used to complement other statistical analysis methods.

The expected risk or baseline probability, denoted by *y*_*n*_ in Eq (7) used to generate the appropriate VLAD chart is an estimated risk that is applied to all cases. In relation to health industries, there are several ways of calculating the baseline probability. Some papers use a risk model derived by other research as the basis for the baseline probability [9, 18, 21] while others use a logistic regression model to generate the baseline probability [13, 22, 23]. There is also research that uses a trial period to obtain the baseline data necessary to generate the baseline probability [19].

### Measuring the performance of passive interactions

In this paper we adapt the cumulative sum methodology, specifically the VLAD methodology to illustrate the performance of candidates’ FP. Thus the objective of IntS is to monitor the strength of interactions, in particular to monitor the strength of passive responses $O^{i}$ given the number of posts $P^{i}$ posted by the candidate *F*^*i*^. If $O_{d}^{i}$ is the observed passive reaction attained by candidate *F*^*i*^ on day *d*, and $B_{d}$, the baseline probability for day *d*, for example, the probability of a candidate’s daily posts getting more likes than the average number of likes for the day, the equation used to calculate the passive interaction strength P_IntS for a candidate over the campaign period is,

$$\begin{array}{c} P\_IntS\left(F^{i}\right)=\sum_{d=1}^{k}\left(B_{d}-O_{d}^{i}\right), \end{array}$$ (8)

with *k* the total number of days in the campaign period. Note that the observed passive reaction depends on the number of likes gathered on the day in question, but is not the bare count of likes. In the language of VLAD, the baseline probability is the expected outcome for the FP of the candidate for the day. With regards to election campaigning, this would be more of a desired outcome rather than an expected outcome, and we prefer to use this former word, as, ultimately election campaigning is about publicising your message above those of your opponents. Thus, P_IntS measures the difference between the desired outcome and the actual, observed outcome.

As Pagel [24] points out, when using control charts to measure performance, it is essential that an appropriate baseline is defined. Hence, the baseline probability, $B_{d}$, used in the calculation of P_IntS needs to reflect the relationship that exists between the posting of the MGE13 candidates and the acquired likes given by the users to the posts. Eq (6) of the regression model indicates that a post posted by a candidate in MGE13 should acquire at least 31 likes. Thus our analysis starts with using the value given by the regression model as the baseline probability. Unfortunately, four out of the 51 FP used during the MGE13 had been deleted within the first week after the election ended, and because the date and time of the posts could not be determined, we had to excluded these 4 FP owned by Gary Lim (*gary*), Azmin Ali (*azmin*), Dzulkefly Ahmad (*dzulkefly*) and Sivarasa Rasiah (*sivarasa*) from the following analysis.

### Using the regression model to calculate the baseline probability

Since $L^{i}$ is the number of likes acquired by *F*^*i*^ over the entire campaign period, let $L_{d}^{i}$ be the number of likes acquired by candidate *F*^*i*^ on day *d*.

According to Eq (6), a post in the MGE13 campaigning should incite at least 31 likes. Thus, the baseline probability, denoted by B(31), is the probability of a candidate’s post acquiring at least 31 likes, regardless of the day the posts are posted. Dividing the number of likes acquired on a particular day $L_{d}^{i}$ by the number of posts posted on that day $P_{d}^{i}$ gives the average number of likes acquired by a post posted on that particular day. At this point we are only looking at the likes gathered each day, so we do not consider the likes gathered by each post on each day as that would unnecessarily complicate the issue.

If *B*1 is the set of all FP that gathered at least 31 likes on average for each post posted, that is, $B1=\{F^{i}:\frac{L^{i}}{P^{i}}\geq 31,1\leq i\leq n\}$ and *B*2 the set of candidates whose FP did not manage this, that is, $B2=\{F^{i}:\frac{L^{i}}{P^{i}}<31,1\leq i\leq n\}$, where *n* is the number of candidates, then the baseline probability B(31) is the probability of a post by candidate *F*^*i*^ getting at least 31 likes, and is given by

$$\begin{array}{c} B\left(31\right)=\frac{\left|B1\right|}{n}. \end{array}$$ (9)

For the MGE13 data, the probability that a candidate’s post is able to generate at least 31 likes is 0.5171 across the campaign period, and thus the baseline probability B(31)=0.5171. This baseline probability is the same for every day of the campaigning period as we have calculated the baseline for the entire campaigning period.

### Using the mean of likes to calculate the baseline probability

However as we can see from the regression model in Fig 2, a large number of FP got many more or far fewer likes than that given by Eq (6). Given the variability in likes gathered on different days (see Fig 3), a better baseline could be the average number of likes gathered on each day. Hence we use the probability of each candidate getting at least as many likes each day as the average number of likes for the day, as the probability baseline, denoting it $B_{d}(\text{likes})$.

Here, instead of having one standard baseline B for the calculation of the P_IntS scores over the entire campaigning period, we use a set of baseline probabilities $B_{d}$ that covers each of the 33 days of campaigning since the number of likes acquired by the FP of the MGE13 candidates varies from day to day (see Fig 3).

Since $L^{i}$ is the number of likes acquired by *F*^*i*^ over the entire campaign period and $L_{d}^{i}$ the number of likes acquired by candidate *F*^*i*^ on day *d*, let $m_{d}^{l}$ be the average number of likes acquired by all candidates on day *d*,

$$\begin{array}{c} m_{d}^{l}=\frac{\sum_{i=1}^{n}L_{d}^{i}}{n}, \end{array}$$ (10)

and $M^{l}$ the set of all average likes over the entire campaign period of *k* days, that is $M^{l}=\left\{m_{d}^{l}:1\leq d\leq k\right\}$.

If $B_{1}^{d}$ is the set of all candidates *F*^*i*^ whose FP gathered more likes than the average for day *d*, that is, $B_{1}^{d}=\left\{F^{i}:L_{d}^{i}\geq m_{d}^{l},1\leq i\leq n\right\}$ and $B_{2}^{d}$ the set of candidates that did not manage this, that is $B_{2}^{d}=\left\{F^{i}:L_{d}^{i}<m_{d}^{l},1\leq i\leq n\right\}$, then the baseline probability $B_{d}(\text{likes})$ is the probability of *F*^*i*^ getting at least the average number of likes on day *d* and is given by

$$\begin{array}{c} B_{d}\left(likes\right)=q_{d}=\frac{|B_{1}^{d}|}{n}. \end{array}$$ (11)

Table 1 shows a sample of $B_{d}(\text{likes})$ for the MGE13 data. Since the likes gathered each day varies, so does the baseline probability (the desired outcome).

**Table 1:** Sample of baseline probability, $B_{d}(\text{likes})$ for MGE13.

Date *d*	$B_{d}(\text{posts})$
3/04/2013	0.2553
4/04/2013	0.1702
⋮	⋮
4/05/2013	0.1702
5/05/2013	0.2340

### Using mean of both posts and likes as the baseline probability

Both previous calculations of baseline probabilities ignore the importance of posts in inciting likes. As Fig 3 shows, there is variability in the number of posts posted on different days of the campaign. Thus, the inclusion of both the mean of the posts as well as the likes in the calculation of the baseline probability should result in these characteristics being reflected in the P_IntS chart. Thus in addition to calculating the $m_{d}^{l}$ as per Eq (10) we calculate the related daily mean of the number of posts, $m_{d}^{p}$.

As mentioned before, given that $P^{i}$ is the number of posts posted by candidate *F*^*i*^ over the entire campaign period, let $P_{d}^{i}$ be the number of posts posted by candidate *F*^*i*^ on day *d* and $m_{d}^{p}$ the average number of posts posted by all candidates on day *d*, then

$$\begin{array}{c} m_{d}^{p}=\frac{\sum_{i=1}^{n}P_{d}^{i}}{n}. \end{array}$$ (12)

Let $M^{p}$ be the set of all average posting numbers over the entire campaign period of *k* days, $M^{p}=\left\{m_{d}^{p}:1\leq d\leq k\right\}$.

To get the baseline probability in this case, we first need to get the posting probability of the candidates. Let $A_{1}^{d}=\left\{F^{i}:P_{d}^{i}\geq m_{d}^{p},1\leq i\leq n\right\}$, $A_{2}^{d}=\left\{F^{i}:0<P_{d}^{i}<m_{d}^{p},1\leq i\leq n\right\}$, $A_{3}^{d}=\left\{F^{i}:P_{d}^{i}=0,1\leq i\leq n\right\}$, that is $A_{1}^{d}$ is the set of all candidates who posted at least as many posts as the average number on the given day, while $A_{2}^{d}$ is the set of those candidates who posted at least one but less than the average number of posts on the given day, and $A_{3}^{d}$ contains candidates who did not post on the given day.

Let $p_{d}=\left|A_{1}^{d}\right|/n$ while $s_{d}=\left|A_{2}^{d}\right|/n$ then the posting probability of *F*^*i*^ on day *d* is given by

$$\begin{array}{c} pr\left(F_{d}^{i}\left(p\right)\right)=\{\begin{array}{cc} p_{d} & \text{if}\;\;P_{d}^{i}\geq m_{d}^{p},\\ s_{d} & \text{if}\;\;0<P_{d}^{i}<m_{d}^{p},\\ 1-(p_{d}+s_{d}) & \text{if}\;\;P_{d}^{i}=0. \end{array} \end{array}$$ (13)

The probability of *F*^*i*^ getting at least as many likes as the average number on day *d*, that is $pr(F_{d}^{i}\left(l\right))$ is as given by Eq (11).

The baseline probability for day *d* is then given by $B_{d}\left(posts\right)=Pr\left(B|A\right)$, where *B* is the probability of a candidate’s posts acquiring at least as many likes as the mean number of likes, conditional on *A*, the probability of the candidate posting at least one post daily. Thus

$$\begin{array}{ccc} B_{d}\left(posts\right) & = & [\left(pr\left(F_{d}^{i}\left(l\right)\right)=q_{d}\right)|\left(pr\left(F_{d}^{i}\left(p\right)\right)=p_{d}\right)\\ & & \;\text{OR}\;\left(pr\left(F_{d}^{i}\left(l\right)\right)=q_{d}\right)\left|\left(pr\left(F_{d}^{i}\left(p\right)\right)=s_{d}\right)\right]\\ & = & q_{d}\times \left(p_{d}+s_{d}\right)\;\text{.} \end{array}$$ (14)

Table 2 shows a sample of the results of the above equation (Eq (14)) for MGE13.

**Table 2:** Sample of baseline probability, $B_{d}(\text{posts})$ for MGE13.

Date *d*	$B_{d}(\text{posts})$
3/04/2013	0.1249
4/04/2013	0.1014
⋮	⋮
4/05/2013	0.1593
5/05/2013	0.1544

We can now describe the process of calculating the passive interaction scores and drawing the resultant P_IntS chart for each FP.

## Results

Once the baseline probability has been calculated and determined, constructing the P_IntS chart goes through 3 stages. In general these stages are, firstly, substituting the observed outcomes with appropriate values (0 or 1), secondly, getting the IntS scores by subtracting the observed outcome from the desired outcome (baseline probability), and finally accumulating and plotting the IntS scores onto the P_IntS chart.

### Substitution of the observed outcomes

For P_IntS(31) (P_IntS(likes)), 0 is the substituted value for observed outcomes whenever the number of likes acquired by the FP is at least 31 (respectively at least $m_{d}^{l}$). For other cases, the substituted value is 1.

Specifically for P_IntS(posts), the substitution of the observed outcomes is based on the following criteria: -1 is given to the observed outcome of a candidate posting at least $m_{d}^{p}$ and acquiring at least $m_{d}^{l}$ likes on day *d*, 0 to the observed outcome of a candidate posting more than 0 but less than $m_{d}^{p}$ yet acquiring at least $m_{d}^{l}$ likes on day *d*, and 1 to the observed outcome of a candidate acquiring less than $m_{d}^{l}$ likes on day *d*. Note that, any candidate who does not post on a particular day automatically gets an observed outcome of 1. Table 3 shows a sample of the P_IntS(posts) substituted observed outcome for MGE13 data.

**Table 3:** A sample of MGE13 after the substitution exercise for P_IntS(posts).

Date *d*	Najib Razak	Chew Hoong Ling	Khairy Jamaluddin
3/04/2013	−1	1	1
4/04/2013	0	1	0
⋮	⋮	⋮	⋮
4/05/2013	−1	1	1
5/05/2013	−1	1	1

-1 indicates that the number of likes is at least $m_{d}^{l}$ with the number of posts at least $m_{d}^{p}$ for that particular day. 0 indicates that the number of likes is at least $m_{d}^{l}$, but the number of posts is more than 0 but less than $m_{d}^{p}$, while 1 indicates that the candidate got less than the average number of likes on that particular day. For example, on 3/04/2013, candidate Najib Razak posted at least the average number of posts which gathered more than the average number of likes getting an observed outcome of -1, while both Chew Hoon Ling and Khairy Jamaluddin either did not post at all or their posts did not attract the desired number of likes, hence both get an observed outcome score of 1.

### IntS = desired outcome - substituted observed outcome

As noted in Eq (8), the P_IntS score for each candidate for each day is the difference between the baseline probability (the desired outcome) and the observed (substituted) outcome for that day. In the first case where we use the regression model and Eq (6) which says that each post in MGE13 must gain 31 likes, the baseline probability, in the case of MGE13, is B(31)=0.5171. Thus the passive interaction score for P_IntS(31) for each candidate for day *d* is given by P_IntS(31)$(F_{d}^{i})=0.5171$ if the candidate’s post on that particular day generates at least 31 likes, otherwise P_IntS(31)$\left(F_{d}^{i}\right)=B\left(31\right)-1=-0.4829$. Thus, a candidate gets a negative P_IntS score every day that his/her FP fails to gain an average of at least 31 likes per post.

In the case of P_IntS(likes), the desired outcome would be the baseline probability $B_{d}$(likes), which varies depending on the day of the campaigning. The values for $B_{d}$(likes) are calculated according to the method given in Eq (11). Thus for each day *d*, the passive interaction score for the candidate’s FP is given by P_IntS(likes)$\left(F_{d}^{i}\right)=B_{d}\left(likes\right)$ if the candidate’s post gathered at least the mean number of likes, otherwise P_IntS(likes)$\left(F_{d}^{i}\right)=1-B_{d}\left(likes\right)$.

When using the variability of the posts along with the acquired likes, the calculation of the passive interaction scores is done slightly differently. Using the substituted observed outcomes as given in Table 3 would result in some daily scores being more than 1. To avoid this and to keep the scores between -1 and 1, the calculation of the P_IntS(posts) scores for each day *d* is done as follows:

$$\begin{array}{c} P\_IntS\left(posts\right)\left(F_{d}^{i}\right)=\{\begin{array}{cc} 1 & \text{if}\;\;P_{d}^{i}\geq m_{d}^{p}\;\&L_{d}^{i}\geq m_{d}^{i},\\ B_{d}\left(posts\right) & \text{if}\;\;0<P_{d}^{i}<m_{d}^{p}\;\&L_{d}^{i}\geq m_{d}^{i},\\ B_{d}\left(posts\right)-1 & \text{if}\;\;L_{d}^{i}<m_{d}^{l}. \end{array} \end{array}$$ (15)

The value of 1 is assigned to cases where the substituted observed outcome (as given in Table 3) is -1 indicating that for that particular day both the number of posts and the number of acquired likes were at least the daily mean of both variables ($P_{d}^{i}$ and $L_{d}^{i}$). This allows us to keep the P_IntS(posts) scores for each day between -1 and 1. Table 4 gives the P_IntS(posts) scores for the sample given in Table 3.

**Table 4:** A sample of MGE13 data *P*_*IntS*_*d*_(posts) scores.

Date *d*	Najib Razak	Chew Hoong Ling	Khairy Jamaluddin
3/04/2013	1.0000	−0.8751	−0.8751
4/04/2013	0.1014	−0.8986	0.1014
⋮	⋮	⋮	⋮
4/05/2013	1.0000	−0.8407	−0.8407
5/05/2013	1.0000	−0.8456	−0.8456

The calculation starts with deducting the substituted observed outcome (as given in Table 3) from the desired outcome ($B_{d}(\text{posts})$). Next the results of the deduction are normalised according to Eq (15) to keep the scores between -1 and 1. Thus on 3/04/2013, candidate Najib Razak gets a P_IntS score of 1, while both Chew Hoong Ling and Khairy Jamaluddin get P_IntS scores for -0.8751, indicating that if they did post on 3/04/2013, their attempt to engage with the public was not very successful.

### Plotting the P_IntS chart

We are now able to plot the passive interaction charts for the various baselines. The passive interaction score for candidate *F*^*i*^ for the entire campaign period, *P*_*IntS*_*F*^*i*^_ is the running total of the scores for the candidate *F*^*i*^ over all the days of the campaign period, $P\_IntS_{F^{i}}=\sum_{d=1}^{k}P\_IntS\left(F_{d}^{i}\right)$. If this cumulative score is greater than zero, then the candidate has elicited more responses from the public than desired, and if it is less than zero, the candidate’s social media presence has not achieved the interaction potential available to him/her.

The P_IntS chart is constructed by plotting the IntS scores over time, which allows the identification of the interactive strength of each candidate’s FP.

## Discussion

As Pagel [24] states, the VLAD chart is very sensitive to the risk estimates used. Thus the selection of an appropriate baseline probability B to be used in calculating the passive interaction scores and drawing the P_IntS chart is important. Table 5 shows the statistical comparison between the P_IntS charts obtained by using the different baselines B described in Section Materials and Methods.

**Table 5:** Comparative statistics of the MGE13 P_IntS scores between P_IntS(posts), P_IntS(likes) and P_IntS(31).

Measurements	P_IntS(posts)	P_IntS(likes)	P_IntS(31)
Range	60.6831	33	33
Max	33	7.9362	17.0638
Min	−27.6831	−25.0638	−15.9362
Std Dev	12.0216	8.1359	7.0647
Median	−7.7954	−7.6809	−0.4146
Mode	−0.8751	−1.5745	−0.4829
Mean	−7.7014	−8.7511	−0.1567
Count	1551	1551	1551

The measurements given in the table include range, standard deviation, median, mode and mean of the 1551 scores for each P_IntS for each day for the 47 MGE13 candidates.

It is important to be able to interpret the results from the P_IntS charts. To enable this, we refer to the description given by Pagel et al. [19] on the results of their VLAD chart that plots the neonatal mortality rate. According to the Pagel et al. [19], if the VLAD score is greater than zero (positive), then there have been fewer deaths than expected and if the score is less than zero (negative), then there have been more deaths than expected. Adapting this interpretation to our data, we define positive IntS scores as indicating strong passive interactions, whereby the number of likes acquired by the FP ID posts is greater than desired. Meanwhile, a negative IntS score points out that the FP ID posts failed to acquire the appropriate number of likes, indicating weak interactions. The movement of the accumulated IntS scores across the chart illustrates the strength of the passive interactions that occurred during the campaigning period. An FP ID that maintains a steady accumulation of positive IntS scores is thus an FP that is performing well.

### Comparison between P_IntS charts for MGE13 data

Looking at the statistical comparison between the different P_IntS (Table 5), we notice that by including the number of the posts in the baseline probability, the range and the standard deviation of the IntS scores starts to spread, giving a much clearer view of the strength of the passive interactions. The negative values of the mean, mode and median for all three P_IntS indicate that the majority of the passive interactions that occurred on the MGE13 candidates’ FP are below zero, alluding to the inability of the FP to maintain a good performance.

**Fig 4:**
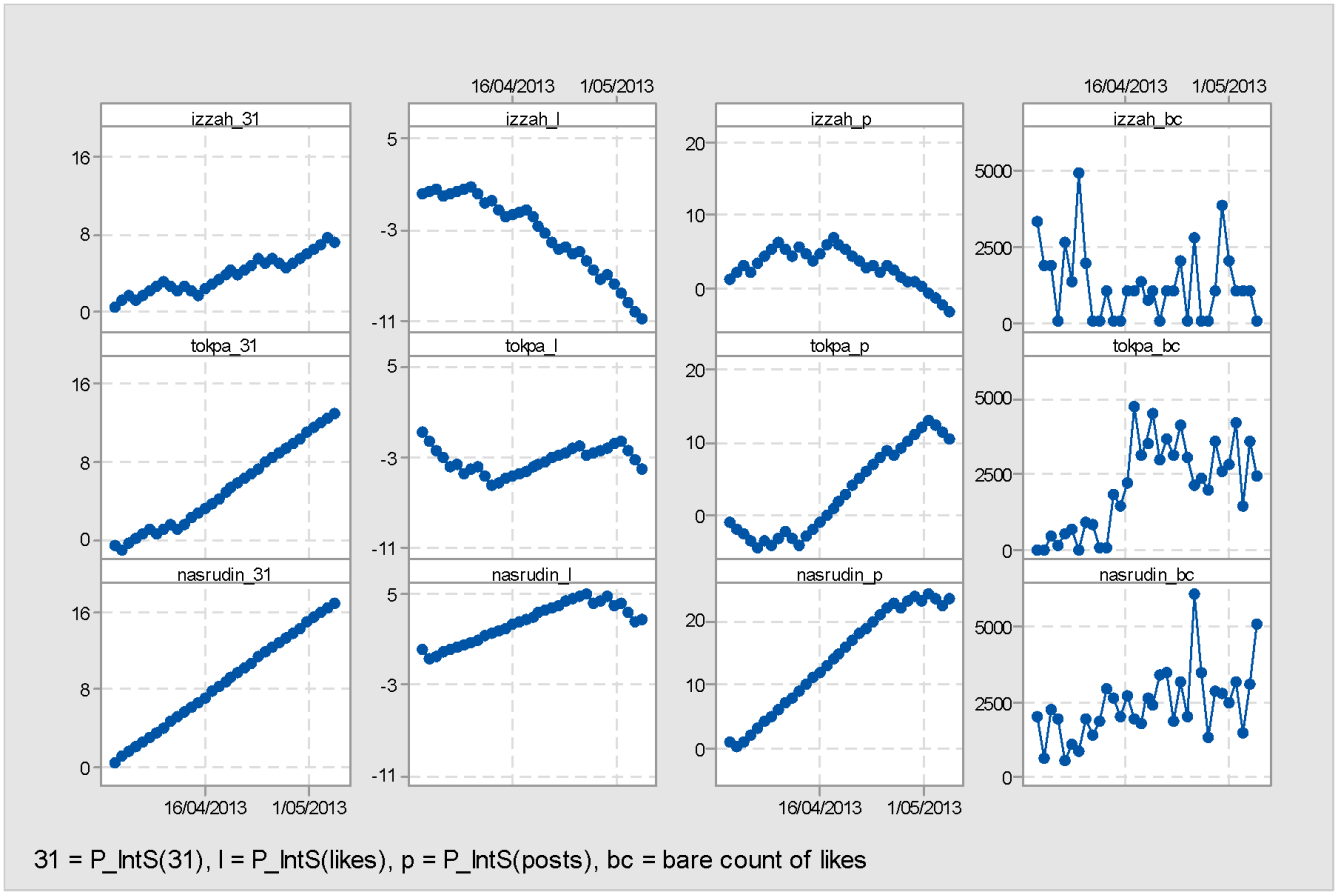
Comparison of P_IntS. Comparison of P_IntS charts for Nurul Izzah, Mustapa Mohamed and Nasrudin Hassan (*izzah, tokpa* and *nasrudin*, respectively). The x-axis for each of the charts ranges over the 33 days of the campaign period, while the y-axis differs depending on the range determined by minimum and maximum scores of each respective P_IntS chart. Each column compares the three selected candidates, with the first row for *izzah*, second row for *tokpa* and the last row for *nasrudin*. The first column compares the P_IntS(31), the second column compares the P_IntS(likes), the third column compares the P_IntS(posts) while the last column shows the bare count of likes for each candidates.

In addition to comparing the statistical results, we compare the P_IntS charts generated from the IntS scores of three sample FP owned by Nurul Izzah (*izzah*), Mustapa Mohamed (*tokpa*) and Nasrudin Hassan (*nasrudin*). The aim here is to highlight the differences further and decide on the best baseline probability B to use to represent the strength of passive interactions in FP campaigning. Fig 4 illustrates the differences between the three different P_IntS charts, for the chosen three FP. All three candidates won their seats, hence the aim is to keep the ‘win’ variable constant and look at the performance of their FP.

According to the MGE13 data, from the bare count point of view (see last column of Fig 4), Mustapa Mohamed and Nasrudin Hassan can be considered both active and popular FP. On the other hand, while Nurul Izzah’s number of posts (209 posts) is slightly higher than the average, the acquired likes (40,627 likes) of her FP was less than average. A good P_IntS chart should illustrate at least this difference between these three candidates, if not more.

P_IntS(31) for all three FP (see first column in Fig 4) shows increasing positive progression across the campaign period. The graph for P_IntS(31) is not very informative as it does not distinguish much between the three candidates, leading to the conclusion that the baseline used, B(31) is inappropriate for discriminating between candidates.

Calculating the IntS scores using only the number of likes, with B(likes) the probability that an FP gained at least the average number of likes each day, as the desired outcome creates a chart, P_IntS(likes) (see column 2 in Fig 4) that also does not clearly illustrate the difference in the interaction strengths of the different FP. As shown in Fig 4, specifically in the second column comparing *izzah_l, tokpa_l* and *nasrudin_l*, the P_IntS(likes) scores for both Nurul Izzah and Mustapa Mohamed show predominantly negative progression, with Nurul Izzah’s final cumulative P_IntS(likes) score being -11. The P_IntS(likes) scores for Nasrudin Hassan show gradual positive progression from 0 with 5 being the maximum attained before another slight dip. Thus P_IntS(likes) goes some way towards distinguishing between the performance of the three candidates’ FP. It shows that Nurul Izzah’s FP is really under-performing. But there is insufficient difference between the other two candidates. The question is whether P_IntS(posts) can do better than this.

Including the variability of posting together with the probability of acquiring likes gives a much better separation of the FP performance, as per the third column comparing *izzah_p, tokpa_p* and *nasrudin_p* in Fig 4. Clearly, Nasrudin Hassan’s P_IntS(posts) scores show a strong upward trajectory rising from 0 to 30, followed by that of Mustapa Mohamed whose final IntS score sits at around 11, with 13 being an interim maximum value. In the case of Nurul Izzah, the progression of the cumulative IntS(posts) scores go from way above 0 (7 being the maximum value) to negative showing the weakening of the FP’s interaction strength towards the end of the MGE13 campaign run.

From the above comparisons (Fig 4), it is clear that the P_IntS(posts) chart is more able to show the variability of passive interactions on FP. The following discussion will thus only consider the findings from the P_Ints(posts) chart.

### Findings from P_IntS(posts) of MGE13 data

**Fig 5:**
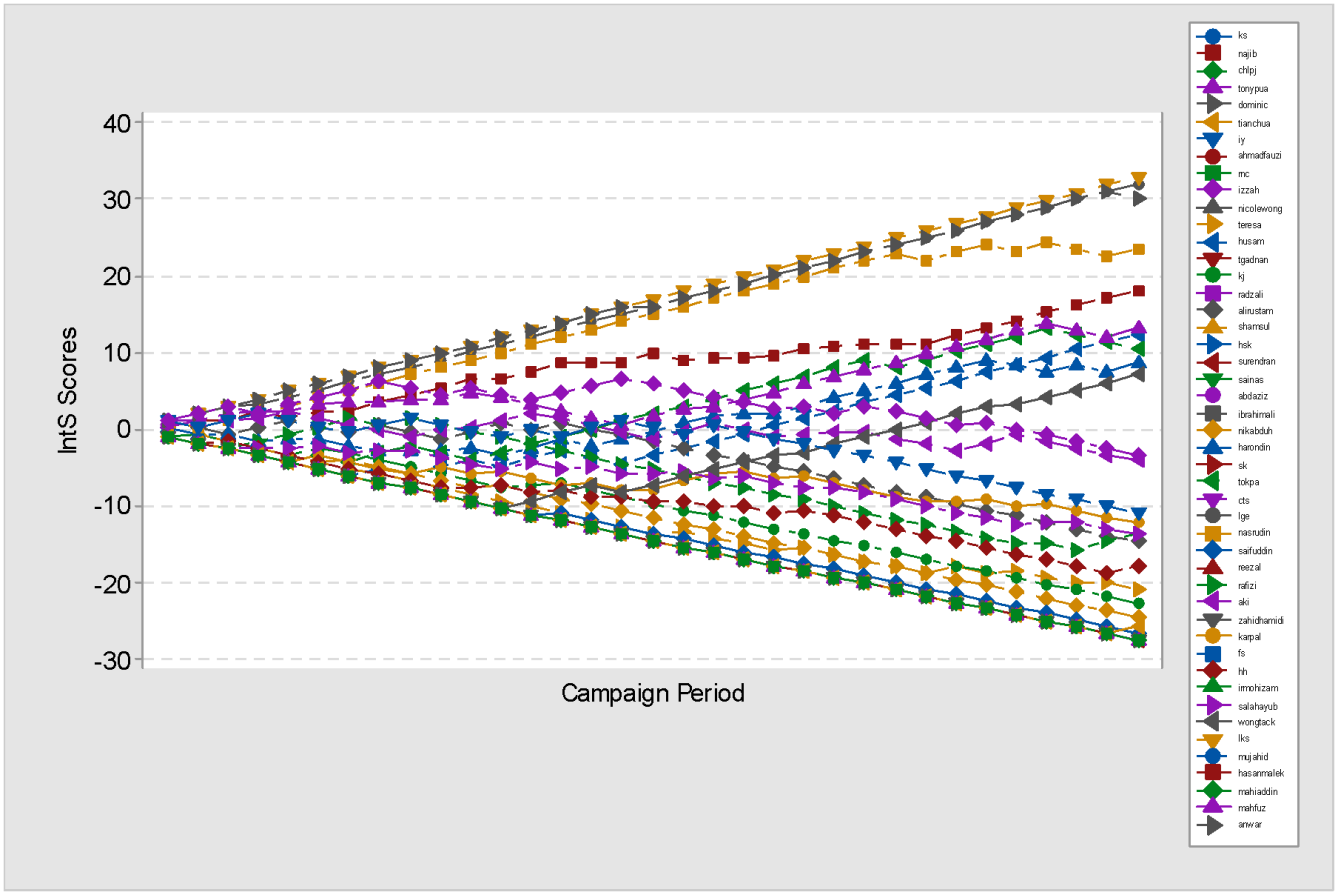
MGE13 P_IntS(posts) chart. P_IntS(posts) chart for MGE13. The y-axis shows the IntS scores and the x-axis indicates the day of the MGE13 campaign. The lines are coloured specific to each of the 47 MGE13 candidates, and drawn based on the accumulated P_IntS scores, while using $B_{d}(\text{posts})$ as the baseline probability. The movement of the lines across the chart illustrates the strength of the passive interactions that occurred during the MGE13 campaigning period.

The passive interaction strength of all the MGE13 FP recorded during the 33 days of campaigning based on P_IntS(posts) is shown in Fig 5.

Reiterating the meaning of positive and negative IntS scores, every increase in the IntS(posts) scores can be taken as an indication of a healthy passive interaction between the posts and the users liking these posts, while troughs or valleys indicate weak passive interactions as a result of the FP not being able to incite the appropriate number of likes either because there was no activity or the posts themselves were not attractive enough. Consistent drop or decrease in IntS scores signals unhealthy passive interactions pointing out that the FP is under-performing.

Fig 5 shows that 28 out of the 47 FP used in generating the P_IntS(posts) chart show a steady progress of negative scores across the campaign period. In a way this indicates that almost 60% of the MGE13 candidates’ FP under-performed. Four FP (as noted previously) were not used in the plotting of the P_IntS charts because the accounts were deleted before we managed to calculate the IntS, as this calculation needed more exact information that was no longer available.

The bare count of likes and posts highlighted Anwar Ibrahim as the *most popular* (443,114 likes), and Nasrudin Hassan as the *most active* (696 posts) (See Fig 1). However, P_IntS(posts) (Fig 5) shows that Lim Kit Siang and Lim Guan Eng are the ones with the *strongest passive interactions* with the Facebook public, with Lim Kit Siang managing to maintain a high rate of increasing and consistently positive P_IntS scores way above the rest of the FP ID, across the campaign period. In addition to that, Fig 5 shows that in the last 10 days of campaigning, the IntS scores of Lim Kit Siang, Lim Guan Eng, Anwar Ibrahim, Nasrudin Hassan and Najib Razak (in that order) are way above 20. This indicates that they have *high performing FPs*.

Among the FP in the top ten most popular and in the top ten most active, three can be seen to have under-performing FP, as indicated by the strength of their passive interactions, in particular during the last 10 days of campaigning. All three of these FP (Fuziah Salleh, Reezal Merican and Ali Rustam) have relatively more posts compared to others, and yet their performance is well below 0.

### Predicting the MGE13 election results

The result of the MGE13 elections for the selected FP candidates used in generating the P_IntS(posts) chart indicates that 66% (31 out of 47) of the candidates won their seats. Only 9 out of that 31 (approximately 29%) candidates managed to incite at least the average accumulated number of likes (49,260 likes) over the entire 33 days of campaigning.

Now, we consider the FP that were ranked as performing well by our method. The P_IntS(posts) chart has 10 FP displaying positive scores on the last day of the campaign (voting day—5/05/2013). It is interesting to note that the P_IntS(posts) scores of these 10 FP (see Table 6) show good variability with none of the FP having the same scores between them. The values range from 7.1544 (Wong Tack) to 33 (Lim Kit Siang). However, out of these 10 FP, 3 candidates (Husam Musa, Haron Din and Wong Tack) lost the election. This, in a way, suggests that the likelihood of a MGE13 candidate with a strong FP interaction strength (positive P_IntS scores) winning an election is approximately 70%. If the prediction were based on the accumulated number of likes acquired for the whole campaign period, all of the 10 MGE13 candidates with strong FP interaction would have won the election, as all of them collected more than the average accumulated number of likes (49,260 likes) over the 33 days of campaigning.

**Table 6:** List of 10 MGE13 candidates with positive P_IntS(posts) scores on day 5/05/2013, together with their election results (Won or Loss) and accumulated likes.

Candidates	P_IntS(posts) Score	Results	Likes
Lim Kit Siang	33.0000	W	223,651
Lim Guan Eng	32.1630	W	376,957
Anwar Ibrahim	30.3282	W	443,114
Nasrudin Hassan	23.7153	W	79,818
Najib Razak	18.2924	W	250,452
Tony Pua	13.1657	W	102,500
Husam Musa	12.4541	L	202,367
Mustapa Mohamed	10.6311	W	69,620
Haron Din	8.6695	L	87,319
Wong Tack	7.1544	L	125,902

Previous studies by Giglietto [5] and Barclay et al. [6] observe that popular candidates (candidates that acquired high number of likes on their FP) are the ones who have won the elections. According to the method used by Giglietto [5], the chances of popular candidate winning the election is 39% while Barclay et al. [6] conclude with the accuracy of 86.6% that there is a strong correlation between the number of likes and the vote share.

As stated before in the Introduction, popularity measurement that is based solely on the number of likes does not present the whole picture. The MGE13 data clearly shows that some popular candidates lost the election. By including the variability of the posts together with the likes over time, P_IntS is more than just a method to predict voting pattern. The objective of P_IntS is to assess the performance of a candidate’s FP during an election campaign.

**Fig 6:**
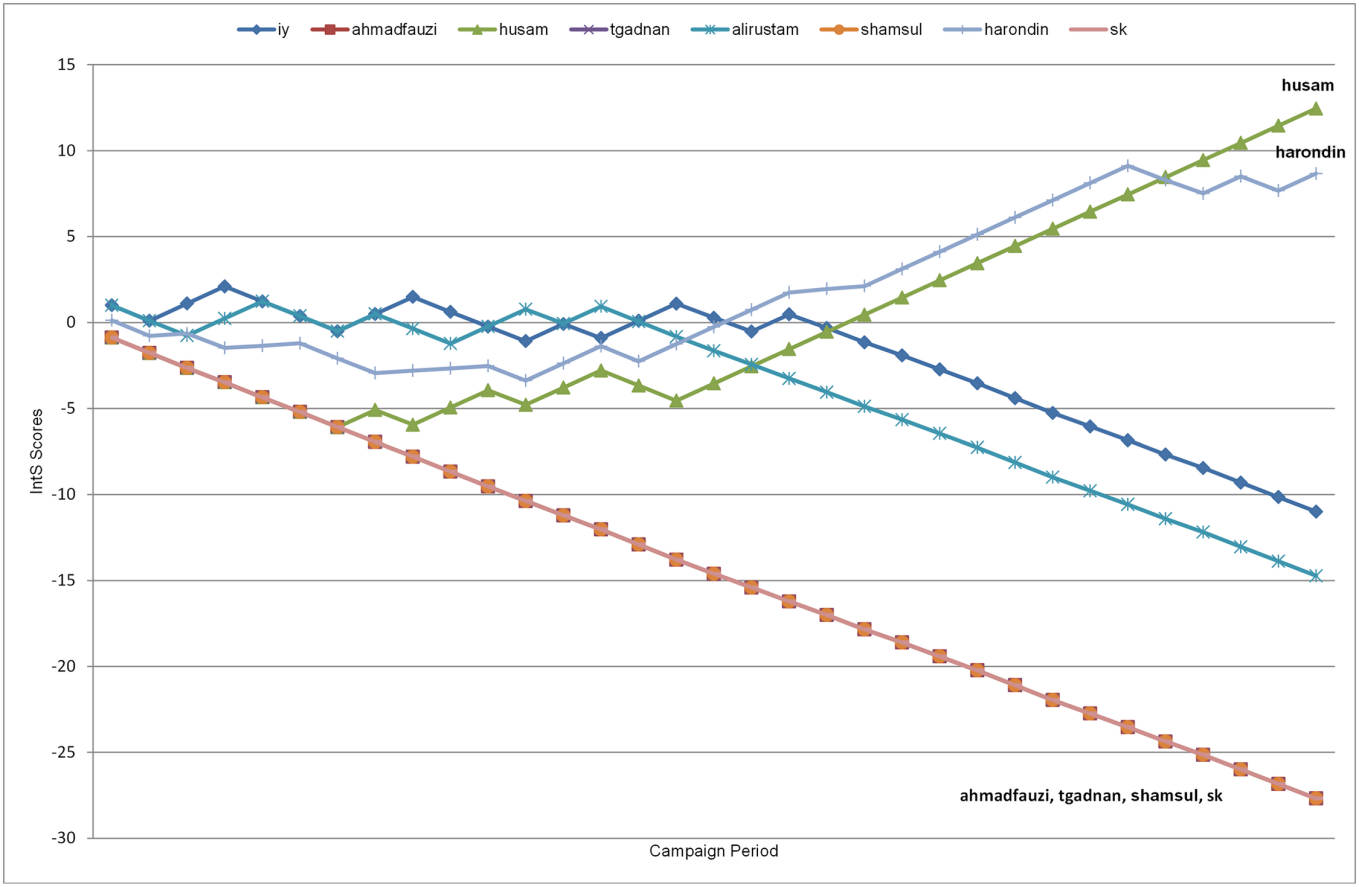
P_IntS(posts): 8 selected candidates. P_IntS(posts) chart for Husam Musa, Tengku Adnan, Haron Din, Shahidan Kassim, Ibrahim Yaacob, Ahmad Fauzi, Ali Rustam and Shamsul Iskandar (*husam, tgadnan, harondin, sk, iy, ahmadfauzi, alirustam* and *shamsul* respectively). The y-axis shows the cumulated P_IntS scores while the x-axis indicates the day of the MGE13 campaign. The lines are coloured based on the 8 candidates mentioned in the legend located at the top of the chart. The chart shows that Husam Musa and Haron Din have the strongest P_IntS, while Ahmad Fauzi, Tengku Adnan, Shamsul Iskandar and Shahidan Kasim shared similar negative progression of P_IntS across the MGE13 campaign.

An interesting angle at this point is comparing the performance of the FP of candidates with respect to their performance in the actual elections. This is especially relevant because, often, popularity in social media is taken to mean success in the election [5, 6]. The results of the MGE13 election show that Husam Musa, Haron Din, Ibrahim Yaacob and Ali Rustam lost to Tengku Adnan, Shahidan Kassim, Ahmad Fauzi and Shamsul Iskandar respectively. By generating a new chart (Fig 6) illustrating the progression of the IntS scores of only these stated FP ID we get an interesting insight into the performance of the FP of these candidates. The performance of Husam Musa, Haron Din, Ibrahim Yaacob and Ali Rustam is clearly much better when compared to their winning opponents. In addition, the performance of Husam Musa and Haron Din jumped up to between 10 and 15 towards the end of the campaign trail. On the other hand, the P_IntS of their winning opponents (Tengku Adnan, Shahidan Kassim, Shamsul Iskandar and Ahmad Fauzi, respectively) share the same steady progression of negative IntS scores across the campaign period. Clearly, other factors are contributing to the winning of seats and having an FP, popular or otherwise is not necessarily enough to win a seat.

From Fig 6 and the accompanying analysis we can conclude that, while on the surface, some FP might be popular or shown to be active (based on the bare count of their likes and posts), based on their interaction strength, the performance of these FP is really below par. As mentioned previously, almost 60% of the FP record a steady progress of negative IntS(posts) scores, signaling that these FP under-performed.

### Comparison with the 2013 Australian Federal Elections (AFE13) data

**Fig 7:**
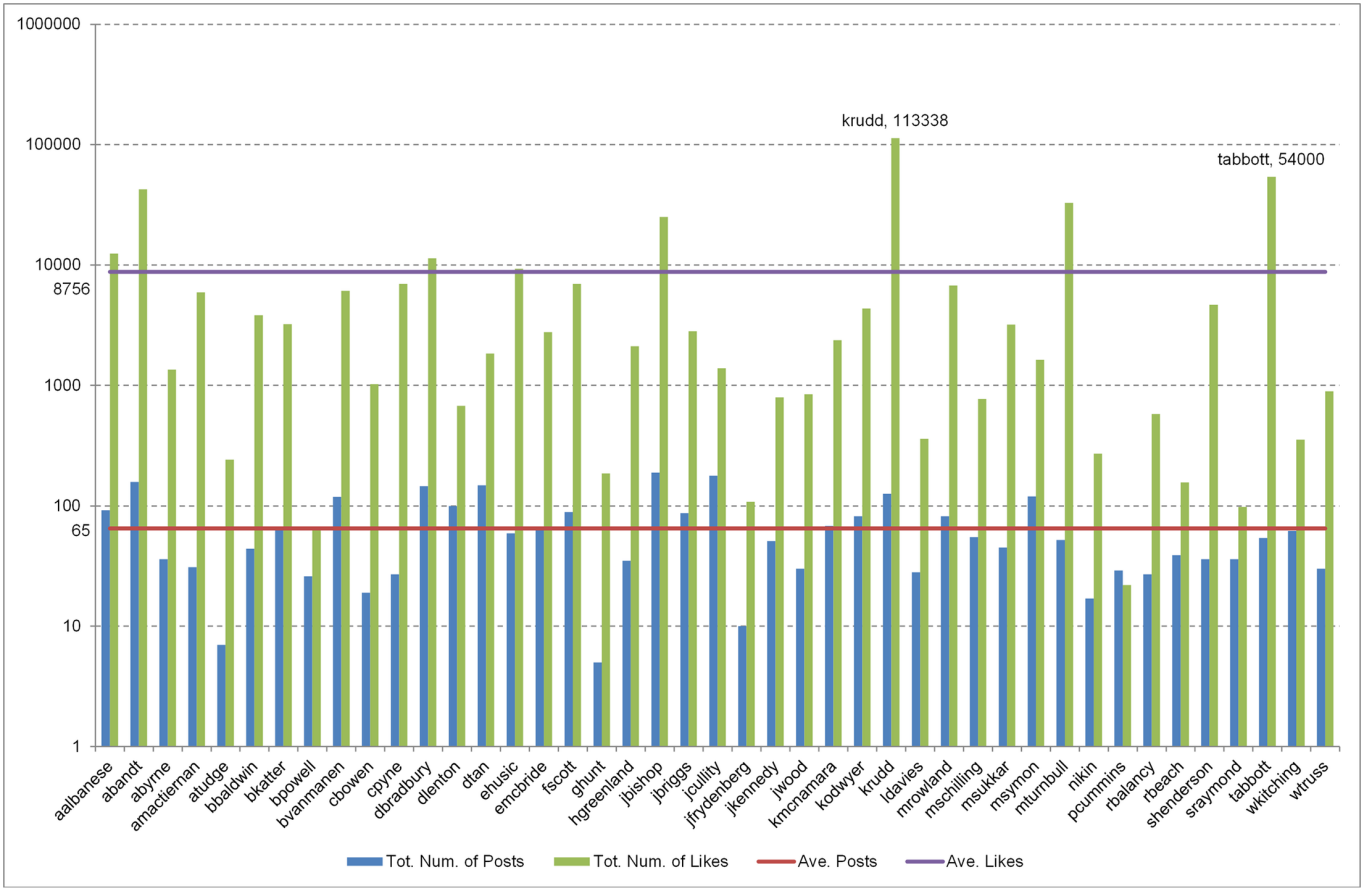
Total *Posts* and *Likes* of the AFE13 FP data. Number of posts (blue bars) and the acquired likes (green bars) recorded on the FP of 43 candidates during the AFE13 campaign. The y-axis shows the log of posts and likes of each candidates as indicated on the x-axis. The purple line indicates the average number of likes received (8,756), while the red line indicates the average number of posts published (65). The graph also shows the total number of likes gained by Kevin Rudd (*krudd*) and Tony Abbott (*tabbott*) over the 33 days of campaigning.

In addition to collecting FP data from the MGE13, we also captured FP data from the 2013 Australian Federal Elections (AFE13) and analysed it for comparison. The AFE13 data encompassing 43 candidates from various parties, shows that during the 35 days of campaigning (4/08/2013 to 7/09/2013), each candidate posted on average 65 posts. Further, on average, each candidate’s FP managed to accumulate 8,756 likes. A total of 2,801 posts were published during the campaign with the number of accumulated likes being 376,519. Kevin Rudd, the incumbent Prime Minister and Leader of the Labor Party managed to gained 113,338 likes with 126 posts, while Tony Abbott, Leader of the Liberal Party, the opposition, acquired 54,000 likes with his 54 posts (see Fig 7). The regression model of the AFE13 ln likes versus ln posts satisfies 36.1% of the AFE13 data with the regression equation predicting that if the AFE13 candidate published one post, the post would acquire approximately 8 likes.

### P_IntS(posts) of AFE13

**Fig 8:**
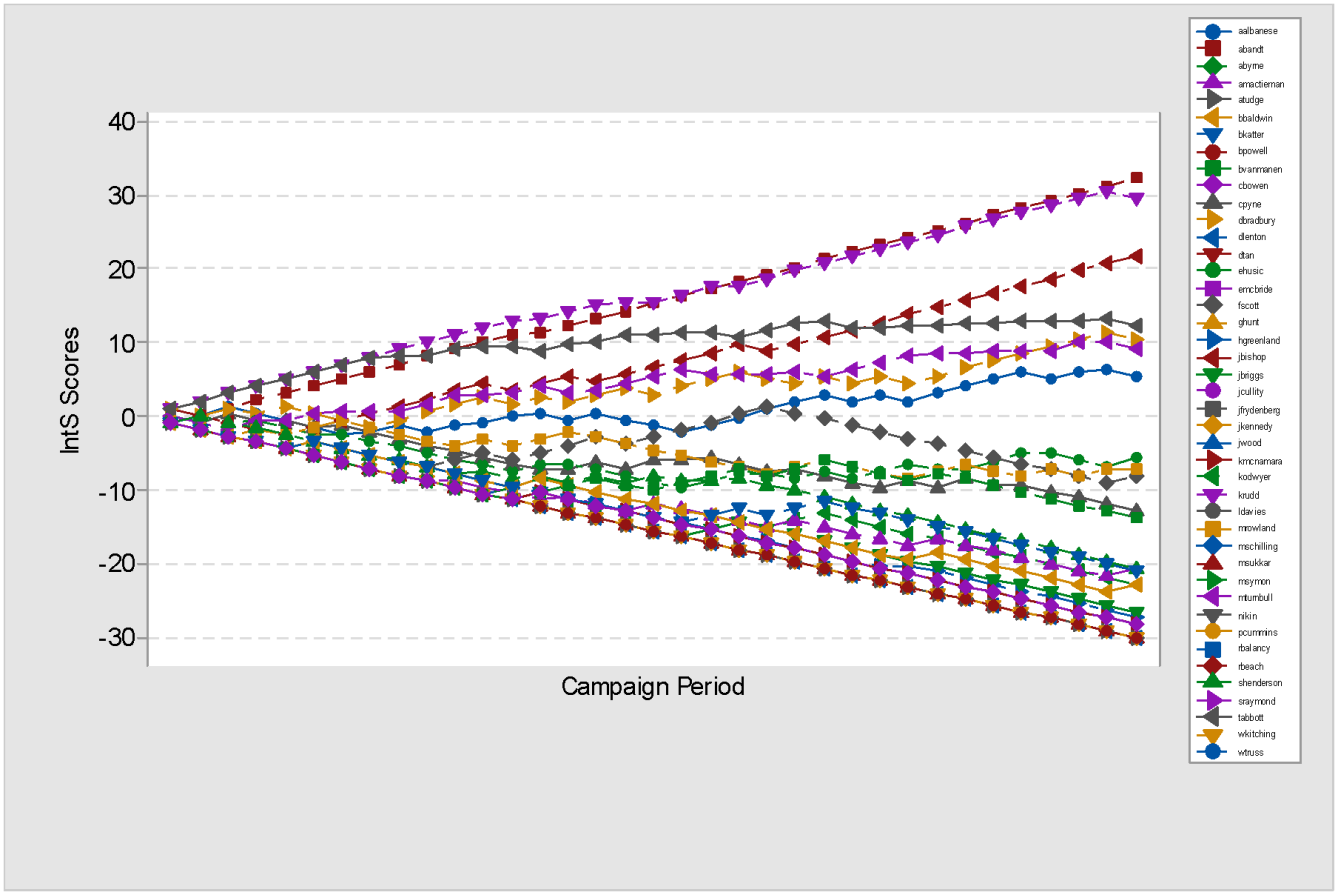
AFE13 P_IntS(posts) chart. P_IntS(posts) chart for AFE13. The y-axis shows the IntS scores and the x-axis indicates the day of the AFE13 campaign. The lines are coloured specific to each of the 43 AFE13 candidates, and drawn based on the cumulated P_IntS(posts) scores, while using $B_{d}(\text{posts})$ as the baseline probability. The movement of the lines across the chart illustrates the strength of the passive interactions that occurred during the AFE13 campaigning period.

The calculation for the baseline probability $B_{d}(\text{posts})$ used in plotting the P_IntS(posts) chart for AFE13 follows similar steps as described previously. The interaction score for day *d* for each of the AFE13 candidates involves deducting the substituted observed outcome from the desired outcome for day *d* (AFE13 $B_{d}(\text{posts})$), and normalising the values according to Eq (15). The AFE13 P_IntS(posts) chart (Fig 8) is based on the cumulative values of the daily AFE13 P_IntS(posts) scores across the 35 days of campaigning.

By and large, the distribution of P_IntS(posts) scores for AFE13 data is quite similar to the distribution of P_IntS(posts) scores for MGE13 (see Table 7). The negative median, mode and mean values for both data indicate that in general, the cumulative P_IntS(posts) scores gained by the examined FP (43 for AFE13 and 47 for MGE13) are below zero signifying weak interactions. Based on the final cumulative P_IntS scores taken on the last day of the AFE13 campaign and Table 8), out of the 43 FP only 16% managed to maintain good interaction across the campaign period.

**Table 7:** Comparative statistics between MGE13 and AFE13 P_IntS(posts) scores.

Measurements	MGE13	AFE13
Range (Max, Min)	60.6831 (33, -27.6831)	60.5960(31.2877, -29.3083)
Standard Deviation	12.0216	−11.2857
Median	−7.7954	−9.7999
Mode	−0.8751	−0.8659
Mean	−7.7014	−9.7122
Count	1551	1462

The table includes the range, standard deviation, median, mode and mean of the P_IntS(posts) scores, respective to each campaign.

**Table 8:** The P_IntS(posts) scores taken at 7/09 for the top 10 FP with the strongest passive interactions, arranged in descending order.

Name	FP ID	P_IntS(posts) Score
Adam Bandt	*abandt*	32.2877
Kevin Rudd	*krudd*	29.7436
Julie Bishop	*jbishop*	21.7258
Tony Abbott	*tabbott*	12.2363
David Bradbury	*dbradbury*	10.5279
Malcolm Turnbull	*mturnbull*	9.1639
Anthony Albanese	*aalbanese*	5.3250
Ed Husic	*ehusic*	−6.7507
Michelle Rowland	*mrowland*	−7.1855
Fiona Scott	*fscott*	−8.1184

Each row presents the name of the candidate, the FP ID and the final cumulative P_IntS(posts) score.

Bare count of likes and posts (see Fig 7) highlight Kevin Rudd as the *most popular* (113,338 likes), and Julie Bishop as the *most active* (189 posts). However, in relation to interaction strength, Fig 8 and Table 8 point out Adam Bandt as the one leading the group, followed by Kevin Rudd and Julie Bishop. All three FP demonstrate a high rate of interaction as evidenced by their final P_IntS(posts) score which are way above those of the rest of the AFE13 FP. Looking at the bare count of likes for Ed Husic and Fiona Scott (listed 9 and 10 in Table 8), imply that the FP are among the most popular, and yet P_IntS(posts) shows that their interaction strength is well below 0.

Similar to the P_IntS(posts) chart for MGE13 (see Fig 5), the P_IntS(posts) chart for AFE13 (see Fig 8) reveals that the performance of many FP deemed to be popular or active (based on bare count likes and posts) is in fact below par. Unlike MGE13, the regression model for AFE13 predicts that a published post will only be able to incite minimal responses (approximately 8 likes) from the users. While this may have to do with the lower population of online users in Australia as opposed to Malaysia, the more reflective measure, the passive interaction score, also showed similar weakness in the AFE13 campaign. Unlike the P_IntS(posts) for MGE13 where about 40% of the FP exhibit strong interactions, the P_IntS(posts) for AFE13 showed that only 16% of the 43 FP used during the campaign experienced positive progression of IntS scores signaling strong interaction strength.

### Conclusion and future work

In this paper we used both the posting variability and the resulting variability in the likes gained by the FP to arrive at a means of measuring the performance of campaigning using FP. As both Figs 4 and 5 as well as the accompanying analysis show, by calculating the probability of a candidate’s posting as well as the probability of the post(s) inciting the appropriate number of likes, it is possible to measure the performance of passive interactions that occur during election campaigning on FP, or on social media in general. The methodology that we have described, besides applying it on the MGE13 and AFE13 data, demonstrates that *P_IntS* is a simple yet informative tool to measure performance.

This paper has shown that the number of likes generated on the candidate’s FP does not depend solely on the number of posts posted, leading to some interesting questions on what makes a post more attractive and able to incite high number of likes. Moreover, is the number of likes really the best variable to use in measuring performance? What about the number of comments? In the future, we hope to further this research by attempting to assess and measure the behaviour of the posts and arrive at some concrete answers on what makes a post tick.
